# Paliperidone ER and oral risperidone in patients with schizophrenia: a comparative database analysis

**DOI:** 10.1186/1471-244X-11-21

**Published:** 2011-02-07

**Authors:** Ibrahim Turkoz, Cynthia A Bossie, Jean-Pierre Lindenmayer, Nina Schooler, Carla M Canuso

**Affiliations:** 1 Johnson & Johnson Pharmaceutical Research and Development, LLC, Titusville, New Jersey, USA; 2 Ortho-McNeil Janssen Scientific Affairs, LLC, Titusville, New Jersey, USA; 3 New York University, New York, New York, USA; 4 SUNY Downstate Medical Center, Brooklyn, New York, USA

## Abstract

**Background:**

To compare the efficacy and tolerability of paliperidone extended-release (ER) with risperidone immediate-release using propensity score methodology.

**Methods:**

Six double-blind, randomized, placebo-controlled, short-term clinical trials for acute schizophrenia with availability of individual patient-level data were identified (3 per compound). Propensity score pairwise matching was used to balance observed covariates between the paliperidone ER and risperidone patient populations. Scores were generated using logistic regression models, with age, body mass index, race, sex, baseline Positive and Negative Syndrome Scale (PANSS) total score and baseline Clinical Global Impressions–Severity (CGI-S) score as factors. The dosage range of paliperidone ER (6-12 mg/day) was compared with 2 risperidone dosage ranges: 2-4 and 4-6 mg/day. The primary efficacy measure was change in PANSS total score at week 6 end point. Tolerability end points included adverse event (AE) reports and weight. AEs with rates ≥5% and with a ≥2% difference between paliperidone ER and risperidone were identified.

**Results:**

Completion rates for placebo-treated subjects in paliperidone ER trials (n = 95) and risperidone trials (n = 122) groups were 36.8% and 51.6%, respectively; end point changes on PANSS total scores were similar (p = 0.768). Completion rates for subjects receiving paliperidone ER 6-12 mg/day (n = 179), risperidone 2-4 mg/day (n = 113) or risperidone 4-6 mg/day (n = 129) were 64.8%, 54.0% and 66.7%, respectively (placebo-adjusted rates: paliperidone ER vs risperidone 2-4 mg/day, p = 0.005; paliperidone ER vs risperidone 4-6 mg/day, p = 0.159). PANSS total score improvement with paliperidone ER was greater than with risperidone 2-4 mg/day (difference in mean change score, -6.7; p < 0.05) and similar to risperidone 4-6 mg/day (0.2; p = 0.927). Placebo-adjusted AEs more common with paliperidone ER were insomnia, sinus tachycardia and tachycardia; more common with risperidone were somnolence, restlessness, nausea, anxiety, salivary hypersecretion, akathisia, dizziness and nasal congestion. Weight changes with paliperidone ER and risperidone were similar (paliperidone ER vs risperidone 2-4 mg/day, p = 0.489; paliperidone ER vs risperidone 4-6 mg/day, p = 0.236).

**Conclusions:**

This indirect database analysis suggested that paliperidone ER 6-12 mg/day may be more efficacious than risperidone 2-4 mg/day and as efficacious as risperidone 4-6 mg/day. The AE-adjusted incidence rates suggest differences between treatments that may be relevant for individual patients. Additional randomized, direct, head-to-head clinical trials are needed to confirm these findings.

## Background

Paliperidone extended-release (ER) is an atypical antipsychotic that delivers the active metabolite of risperidone (9-hydroxyrisperidone) using OROS^® ^technology. This formulation minimizes drug plasma fluctuations relative to oral immediate-release risperidone and eliminates the need for initial dose titration [1,2]. The 2 drugs also differ in that risperidone is metabolized in the liver via the cytochrome (CYP) P450 2D6 pathway, whereas this pathway is minimally involved in the metabolism of paliperidone ER. Therefore, the potential for clinically significant interactions between paliperidone ER and other drugs metabolized by the CYP P450 2D6 pathway may be minimal [3,4].

The efficacy and safety of risperidone for the treatment of schizophrenia were established more than 15 years ago [5,6]. More recently, multinational placebo-controlled studies have shown paliperidone ER 3-15 mg/day to be both efficacious and safe, with discontinuation rates due to adverse events (AEs) similar to placebo [7-9]. To date, and to our best knowledge, however, no studies have been specifically designed to directly compare the efficacy of paliperidone ER and oral risperidone.

The objective of the current analysis was to perform a post hoc statistical indirect comparison of paliperidone ER and risperidone using propensity score matching. Propensity scores, originally introduced by Rosenbaum and Rubin [10], can be used to create treatment groups that are balanced on a large number of baseline characteristics; they are the observational study analogue of randomization.

## Methods

### Analysis sets

This comparative analysis pooled double-blind, randomized, placebo-controlled, short-term (4- to 8-week) clinical studies of paliperidone ER or risperidone monotherapy in acutely ill adults with schizophrenia aged 18-65 inclusive, in which detailed patient-level data were available. A total of 6 studies were identified: 3 for paliperidone ER and 3 for risperidone (Table 1). A literature search (through December 31, 2009) confirmed that these 6 studies were the only studies that met the inclusion criteria and had individual patient-level data that were available. In the risperidone studies (conducted between 1988 and 1996), prior antipsychotic treatment consisted primarily of conventional agents, whereas in the paliperidone ER studies (conducted between 2004 and 2005), over 50% of subjects had received prior treatment with atypical antipsychotics. Therefore, subjects from the paliperidone ER trials were included only if they had received conventional antipsychotics within 90 days before the start of the study, regardless of concomitant use of other agents, including atypical antipsychotics. All subjects in risperidone studies RIS-USA-1 and RIS-INT-3 were included, and RIS-USA-72 subjects were included if they did not take atypical antipsychotics prior to the study (clozapine and risperidone were available at the time of this study). DSM-III-R/IV diagnosis codes for inclusion were 295.10, 295.20, 295.30, 295.60 or 295.90. Exclusions were age >65 years and risperidone >8 mg/day. Because the RIS-USA-1 trial had a flexible-dosing regimen, subjects were assigned to a dose group based on their modal dose. Defined treatment groups were paliperidone ER 6-12 mg/day, risperidone 2-4 mg/day, risperidone 4-6 mg/day or placebo. Since the studies were conducted during different decades, the above inclusion and exclusion criteria for this analysis were chosen to minimize differences between the populations.

**Table 1:** Randomized placebo-controlled studies available for analysis

Paliperidone ER						
**Study** **(Year Completed)**	N*	**Population**	**Dose (mg/day)**	**Duration (Weeks)**	**Efficacy Measures**	**Safety Measures**

PALI-SCH-303 (2005) [7]	500^†^	• Patients with schizophrenia, diagnosis for ≥1 yr • Agreed to hospitalization for at least the first 14 days of the study • Acute episode with PANSS total score 70-120	6, 9, 12 (qd)	6	PANSS, CGI	SAS, BARS, AIMS, AEs, labs, weight
PALI-SCH-304 (2004) [8]	327^†^	• Patients with schizophrenia, diagnosis for ≥1 yr • Agreed to hospitalization for at least the first 14 days of the study • Acute episode with PANSS total score 70-120	6, 12 (qd)	6	PANSS, CGI	SAS, BARS, AIMS, AEs, labs, weight
PALI-SCH-305 (2005) [9]	366^†^	• Patients with schizophrenia, diagnosis for ≥1 yr • Agreed to hospitalization for at least the first 14 days of the study • Acute episode with PANSS total score 70-120	3, 9, 15 (qd)	6	PANSS, CGI	SAS, BARS, AIMS, AEs, labs, weight
**Risperidone**						
**Study**	**N**	**Population**	**Dose (mg/day)**	**Duration (Weeks)**	**Efficacy Measures**	**Safety Measures**
RIS-USA-1 (1990)^‡^	160	• Patients with schizophrenia • Inpatients at the start of the study • Minimum BPRS score of 30, with a minimum score of at least moderate (4) on 2 of the following items: conceptual disorganization, suspiciousness, hallucinatory behavior, unusual thought content	1-10^§ ^(qd)	6	BPRS, CGI	ESRS, AIMS, AEs, labs, weight
RIS-INT-3 (1991) [5]; [6]	523	• Patients with chronic schizophrenia • Inpatients at the start of the study • PANSS total score 60-120	2, 6, 10,16 (bid)	8	PANSS, CGI	ESRS, AEs, labs, weight
RIS-USA-72 (1996)^‡^	246	• Patients with schizophrenia • Inpatients at the start of the study • PANSS total score 80-120, with a PANSS score ≥8 for the sum of 2 of the following items: conceptual disorganization, suspiciousness, hallucinatory behavior, unusual thought content	4, 8 (qd)	4	PANSS	ESRS, AEs, labs, weight

*Intent-to-treat population.

^†^N values represent subjects who received the recommend dose of paliperidone ER (3-12 mg/day) or placebo.

^‡^Data on file.

^§^Flexible dosing.

PANSS = Positive and Negative Syndrome Scale; CGI = Clinical Global Impressions; SAS = Simpson-Angus Scale; BARS = Barnes Akathisia Rating Scale; AIMS = Abnormal Involuntary Movement Scale; AEs = adverse events; BPRS = Brief Psychiatric Rating Scale; ESRS = Extrapyramidal Symptoms Rating Scale.

Paliperidone ER in the dose range 6-12 mg/day was chosen for comparison because it is likely to provide a favorable risk/benefit for most patients with schizophrenia. (The recommended range is 3-12 mg/day.) Based on pharmacokinetic data, paliperidone 6-12 mg/day and risperidone 2-4 mg/day were compared because they were expected to provide similar systemic drug exposure [2]. Paliperidone ER 6-12 mg/day was also compared with risperidone 4-6 mg/day as these dose ranges were expected to yield the most favorable risk/benefit ratios based on clinical data. The placebo groups of the risperidone and paliperidone ER trials were referred to as placebo (risperidone [RIS]) and placebo (paliperidone ER [PALI]). They were pooled for some analyses as described below.

### Efficacy and safety outcomes

Efficacy endpoints included change in the Positive and Negative Syndrome Scale (PANSS) total (primary efficacy measure) [11] and factor scores [12] (for all studies excluding RIS-USA-1), Clinical Global Impressions-Severity (CGI-S) score [13] (for all studies excluding RIS-USA-72), response rates (≥30% decrease in total PANSS score from baseline), weight and spontaneously reported AEs. Owing to differences in AE coding between programs, 2 clinicians reviewed all AEs in a blinded fashion to map verbatim terms to preferred terms; these were then mapped to a System Organ Class (SOC) using MedDRA (Medical Dictionary for Regulatory Activities) terminology. Extrapyramidal symptoms (EPS) measures were not included due to the difference in rating scales between the risperidone (Extrapyramidal Symptoms Rating Scale) and paliperidone (Simpson Angus Scale) studies.

### Statistical analysis

#### Propensity score matching

In the present analysis, propensity score matching was used to create comparison groups that were similar with respect to the distribution of a number of demographic and baseline characteristics so that indirect comparisons would be more valid. A propensity score for each patient was created using variables common to both sets of studies from multiple logistic regression models. Subjects were matched as stringently as possible while maintaining sufficient patient numbers for analysis. A 1-to-many matching scheme with a caliper (distance) of 0.05 was used to match each treated subject with the closest control. In 1-to-many matching, all cases are initially matched to their "best" control in the first iteration, and "next best" matches next in hierarchical sequence until no more matches can be made. Best matches are those with the smallest difference in propensity scores. Goodness of matches and available sample sizes were evaluated to determine the matching algorithm used to create a new analysis population.

Based on the selected population of 933 subjects from 6 placebo-controlled trials, matched cohorts were created by 1) pairing risperidone (regardless of dose) and its corresponding placebo group (placebo [RIS]) and 2) pairing paliperidone ER (regardless of dose) and its corresponding placebo group (placebo [PALI]). This was followed by pairing from the resulting risperidone and paliperidone ER groups identified in steps 1 and 2. All subjects in the matched risperidone/paliperidone ER group were included in the final sample. The placebo groups identified in the pairing with risperidone or paliperidone ER were then compared to confirm whether they could be combined into a pooled placebo group for efficacy analyses. This provided 3 groups (risperidone, paliperidone ER and placebo) for the efficacy comparisons. The dependent variable of the multiple logistic regression models was treatment group, and the independent variables were age, sex, race (white vs all other), baseline body mass index (BMI), baseline CGI and baseline PANSS. Choice of the independent variables was based on clinical relevance and their inclusion in all 6 studies.

#### Analysis methods

The primary comparisons were between paliperidone ER 6-12 mg/day and each dose range of risperidone. Changes from baseline to end point (week 4 in RIS-USA-72, week 8 in RIS-INT-3 and week 6 for all other studies) were evaluated using the last-observation-carried-forward (LOCF) approach. Comparisons were calculated from analysis-of-covariance models, with treatment as the between group factor and baseline as the covariate. Categorical variables and response rates at end point were evaluated using a chi-square test. No adjustments for multiple comparisons were made. Data were analyzed using SAS^® ^(Version 9.1 for Windows).

Differences between placebo groups were examined, and placebo-adjusted rates were reported for those that were significant (as was the case for completion and AE rates). For adjusting AE rates, placebo corrections were applied to each active group (ie, active % – corresponding placebo %) according to the equation: ([risperidone % – placebo (RIS) %] – [paliperidone ER % – placebo (PALI) %]). If the placebo rate for any AE was higher than for active treatment, active treatment minus placebo was set equal to 0. Differences in AEs between paliperidone ER and risperidone were noted when the AE differential was ≥2% after correcting for the placebo rates.

## Results

**Figure 1:**
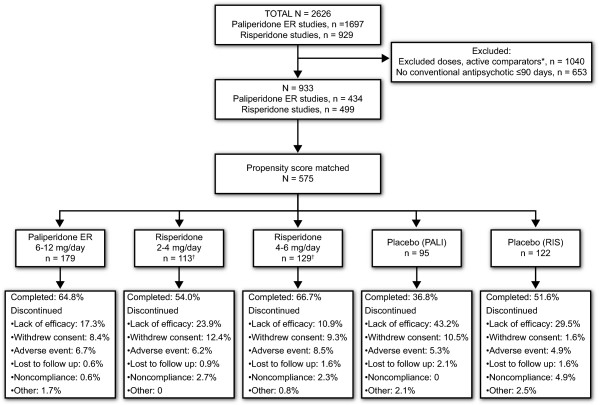
**Patient flow and disposition**. *Subjects who received haloperidol, olanzapine, risperidone >8 mg/day or an unspecified risperidone dose, paliperidone ER 3 or 15 mg/day. ^†^Subjects who received risperidone 4 mg/day (n = 63) were included in both risperidone groups.

Data were available from 2626 subjects in the 6 studies; of these, 933 subjects were retained in the propensity score-matched population (Figure 1). A total of 179 subjects received paliperidone ER 6-12 mg/day, 113 subjects received risperidone 2-4 mg/day, 129 subjects received risperidone 4-6 mg/day, 95 received placebo in paliperidone studies (placebo [PALI]) and 122 received placebo in risperidone studies (placebo [RIS]). Study completion rates for the 2 placebo groups were 36.8% with placebo (PALI) and 51.6% with placebo (RIS) (p = 0.030) (Figure 1). Risperidone subjects receiving 4 mg/day (n = 63) were included in both risperidone groups. A comparison of the placebo (PALI) and placebo (RIS) groups for the purposes of validating the selection criteria used to match the populations indicated that baseline demographic and clinical characteristics, and between-group differences at end point on PANSS total and factor change scores (all p values > 0.05), were similar (Table 2). Therefore, the placebo groups were pooled (n = 217) for further efficacy analyses.

**Table 2:** Baseline parameters and efficacy outcomes in placebo groups from paliperidone ER and risperidone studies

Baseline Demographic and Clinical Characteristics	Placebo (PALI) n = 95	Placebo (RIS) n = 122	p Value
Age, years, mean (SD)	36.7 (10.9)	38.0 (10.1)	0.338

Female, n (%)	23 (24.2)	18 (14.8)	0.083

Race, n (%)			
Caucasian	65 (91.6)	73 (93.6)	0.758
Other	6 (8.5)	5 (6.4)	

BMI, mean (SD), kg/m^2^	24.9 (5.2)	25.3 (3.2)	0.538

PANSS total score, mean (SD)	94.5 (12.2)	92.6 (12.3)	0.254

CGI-S, score (SD)	4.6 (0.7)	4.7 (0.8)	0.105

**PANSS, Adjusted Mean Change (SE) at End Point**	**Placebo** **(PALI)** **n = 92**	**Placebo** **(RIS)** **n = 94**	**p Value**

Total score	-6.5 (2.5)	-5.4 (2.5)	0.768

Factor score			
Negative	-1.3 (0.7)	-2.3 (0.7)	0.280
Positive	-2.5 (0.8)	-1.6 (0.8)	0.423
Anxiety/depression	-1.3 (0.4)	-1.8 (0.4)	0.402
Disorganized thoughts	-1.2 (0.6)	-0.6 (0.6)	0.507
Uncontrolled hostility/excitement	0.4 (0.5)	0.9 (0.5)	0.530

PALI = paliperidone ER; RIS = risperidone; BMI = body mass index; PANSS = Positive and Negative Syndrome Scale; CGI-S = Clinical Global Impressions—Severity.

p values are from ANOVA models with factor treatment for continuous variables and chi-square test for categorical variables.

### Comparison of paliperidone ER and risperidone groups

Baseline demographic and clinical characteristics in the paliperidone ER and risperidone dose groups were comparable, as expected, because of propensity score matching (Table 3). Completion rates were 64.8% with paliperidone ER 6-12 mg/day, 54.0% with risperidone 2-4 mg/day and 66.7% with risperidone 4-6 mg/day. Placebo-adjusted rates were 28.0%, 2.4% and 15.1% in the paliperidone ER, risperidone 2-4 mg/day and risperidone 4-6 mg/day groups, respectively (placebo-adjusted p values for paliperidone ER vs risperidone 2-4 mg/day, p = 0.005; paliperidone ER vs risperidone 4-6 mg/day, p = 0.159) (Figure 1).

**Table 3:** Baseline characteristics in the propensity score-matched paliperidone ER, risperidone and pooled placebo groups

	Paliperidone ER 6-12 mg/day n = 179	Risperidone 2-4 mg/day n = 113	Risperidone 4-6 mg/day n = 129	Pooled Placebo n = 217
Age, years, mean (SD)	37.4 (11.3)	37.8 (10.6)	37.1 (10.1)	37.4 (10.4)

Female, n (%)	45 (25.1)	31 (27.4)	32 (24.8)	41 (18.9)

Race, n (%)				
Caucasian	136 (76.0)	83 (73.5)	99 (76.7)	138 (63.6)
Other	43 (24.0)	30 (26.5)	30 (23.3)	79 (36.4)

BMI, mean (SD), kg/m^2^	25.2 (4.7)	25.5 (3.3)	25.5 (3.4)	25.1 (4.2)

PANSS total score, mean (SD)	94.3 (11.9)	94.4 (15.2)	96.2 (16.6)	93.4 (12.2)

CGI-S score, mean (SD)	4.8 (0.7)	4.8 (0.6)	4.8 (0.7)	4.7 (0.8)

BMI = body mass index; PANSS = Positive and Negative Syndrome Scale; CGI-S = Clinical Global Impressions—Severity.

All the p values are >0.05 for the group comparisons. p values are from ANOVA models with factor treatment for continuous variables and chi-square test for categorical variables.

### Comparison of paliperidone ER 6-12 mg/day and risperidone 2-4 mg/day

Paliperidone ER showed greater improvement at end point on the mean PANSS total score compared with risperidone 2-4 mg/day (difference in mean change score, -6.7; p < 0.05), and more improvement on the PANSS factor scores for negative symptoms (difference in mean change score, -2.1; p < 0.05) (Table 4). Paliperidone ER, but not risperidone 2-4 mg/day, was superior to combined placebo on the PANSS total score and factor scores for negative symptoms, anxiety/depression and disorganized thoughts (p < 0.05 for all comparisons of paliperidone ER vs placebo). A similar pattern was observed with the CGI-S score; improvement was greater with paliperidone ER than risperidone 2-4 mg/day (p < 0.001) or combined placebo, but not with risperidone 2-4 mg/day vs combined placebo (Table 4). The percentage of subjects who achieved a response (≥30% decrease in total PANSS score from baseline) was 55.1% with paliperidone ER vs 40.7% with risperidone 2-4 mg/day (p < 0.05). Response rates with paliperidone ER and risperidone 2-4 mg/day were both significantly higher than with combined placebo (Table 4).

**Table 4:** Efficacy findings for paliperidone ER and risperidone dose groups (change from baseline to end point)

	Paliperidone ER 6-12 mg/day n = 179	Risperidone 2-4 mg/day n = 113	Risperidone 4-6 mg/day n = 126*	Pooled Placebo n = 186*
**PANSS**				

Total score, adjusted mean change (SE)	-18.4 (1.7)	-11.6 (2.2)	-18.7 (2.0)	-6.4 (1.7)

Mean difference (estimated SE)^†^				
Active-PBO	-12.0 (2.4)^‡^	-5.4 (2.8)	-12.0 (2.6)^‡^	NA
PALI-RIS 2-4 mg	-6.7 (2.8)^§^	NA	NA	NA
PALI-RIS 4-6 mg	0.2 (2.6)	NA	NA	NA

Negative factor, adjusted mean change (SE)	-4.7 (0.5)	-2.5 (0.6)	-3.8 (0.5)	-2.1 (0.4)

Mean difference (estimated SE)^†^				
Active-PBO	-2.6 (0.6)^‡^	-0.5 (0.8)	-1.7 (0.7)^§^	NA
PALI-RIS 2-4 mg	-2.1 (0.8)^§^	NA	NA	NA
PALI-RIS 4-6 mg	-0.9 (0.7)	NA	NA	NA

Positive factor, adjusted mean change (SE)	-6.2 (0.5)	-4.5 (0.7)	-6.3 (0.7)	-2.3 (0.5)

Mean difference (estimated SE)^†^				
Active-PBO	-4.0 (0.8)^‡^	-2.2 (0.9)^§^	-4.1 (0.8)^‡^	NA
PALI-RIS 2-4 mg	-1.7 (0.9)	NA	NA	NA
PALI-RIS 4-6 mg	0.1 (0.8)	NA	NA	NA

**Anxiety/depression, adjusted mean change (SE)**	-2.3 (0.3)	-1.7 (0.3)	-2.5 (0.3)	-1.4 (0.3)

Mean difference (estimated SE)^†^				
Active-PBO	-0.9 (0.4)^§^	-0.3 (0.4)	-1.1 (0.4) ^§^	NA
PALI-RIS 2-4 mg	-0.6 (0.4)	NA	NA	NA
PALI-RIS 4-6 mg	0.2 (0.4)	NA	NA	NA

**Disorganized thoughts, adjusted mean change (SE)**	-3.5 (0.4)	-2.3 (0.5)	-4.2 (0.5)	-1.1 (0.4)

Mean difference (estimated SE)^†^				
Active-PBO	-2.4 (0.6)^‡^	-1.3 (0.7)	-3.2 (0.7)^‡^	NA
PALI-RIS 2-4 mg	-1.1 (0.7)	NA	NA	NA
PALI-RIS 4-6 mg	0.8 (0.7)	NA	NA	NA

**Uncontrolled hostility/excitement, adjusted mean change (SE)**	-1.4 (0.3)	-0.6 (0.4)	-2.0 (0.4)	0.6 (0.3)

Mean difference (estimated SE)^†^				
Active-PBO	-2.0 (0.5)^‡^	-1.2 (0.5)^§^	2.6 (0.5)^‡^	NA
PALI-RIS 2-4 mg	-0.8 (0.5)	NA	NA	NA
PALI-RIS 4-6 mg	0.6 (0.5)	NA	NA	NA

**CGI-S, adjusted mean change (SE)^¶^**	-0.9 (0.1)	0.0 (0.2)	-0.1 (0.2)	-0.2 (0.1)

Mean difference (estimated SE)^†^				
Active-PBO	-0.7 (0.1)^‡^	0.2 (0.2)	0.0 (0.3)	NA
PALI-RIS 2-4 mg	-0.9 (0.2)^‡^	NA	NA	NA
PALI-RIS 4-6 mg	-0.8 (0.3)^§^	NA	NA	NA

**Response, %^#^**	55.1	40.7	51.6	28.1

Active-PBO	26.9^§^	28.5^§^	23.4^§^	NA
PALI-RIS 2-4 mg	14.4^§^	NA	NA	NA
PALI-RIS 4-6 mg	3.5	NA	NA	NA

Separate models were run for comparison of risperidone 2-4 mg/day and risperidone 4-6 mg/day against paliperidone ER 6-12 mg/day and pooled placebo groups. Adjusted means for the pooled placebo group are presented from the model comparing paliperidone ER with risperidone 4-6 mg/day. Data are nearly identical to those for the model comparing paliperidone ER with risperidone 2-4 mg/day. p values are from ANCOVA models with factor treatment and covariate baseline score for continuous variables and chi-square test for categorical variables.

*Not all subjects had a PANSS assessment.

^†^A negative sign indicates greater improvement vs comparator.

^‡^p < 0.0001; ^§^p < 0.05.

^¶^Numbers of subjects assessed were 179, 53, 69 and 167 for the paliperidone ER, risperidone 2-4 mg/day, risperidone 4-6 mg/day and pooled placebo groups, respectively.

^#^Response was defined as ≥30% decrease in total PANSS score from baseline.

PANSS = Positive and Negative Syndrome Scale; PALI = paliperidone ER; RIS = risperidone PBO = placebo; CGI-S = Clinical Global Impressions—Severity; NA = not applicable.

### Comparison of paliperidone ER 6-12 mg/day and risperidone 4-6 mg/day

Changes in mean PANSS total score and all factor scores did not differ significantly (all between-group p values > 0.05); both paliperidone ER and risperidone 4-6 mg/day were superior to combined placebo on these measures (all p values vs placebo < 0.05). Improvement on the CGI-S scale was greater with paliperidone ER than risperidone 4-6 mg/day (p < 0.05); paliperidone ER, but not risperidone 4-6 mg/day, was superior to combined placebo (p < 0.001). Response rates with paliperidone ER and risperidone 4-6 mg/day were comparable, and rates with both active treatments were higher than with combined placebo (p < 0.001 for both active treatments vs combined placebo) (Table 4).

### Safety

No statistical tests were applied to AE data, but placebo corrections were applied to each active group as described in the Methods section. Placebo-adjusted AE rates that differed by ≥ 2% between groups are presented (Table 5). For the paliperidone ER vs risperidone 2-4 mg comparison, placebo-adjusted AEs more common with paliperidone ER than risperidone were sinus tachycardia and tachycardia; placebo-adjusted AEs more common with risperidone than paliperidone ER were somnolence, restlessness, nausea, anxiety, salivary hypersecretion and akathisia (Table 5). For paliperidone ER vs risperidone 4-6 mg, placebo-adjusted AEs more common with paliperidone ER than risperidone included insomnia and sinus tachycardia; placebo-adjusted AEs more common with risperidone than paliperidone ER were somnolence, restlessness, nausea, anxiety, salivary hypersecretion, akathisia, nasal congestion and dizziness (Table 5).

**Table 5:** Treatment-emergent adverse events in ≥5% in any active-treatment or placebo groups

						Placebo-Adjusted Difference* ≥2%	
						
	**Paliperidone ER** **6-12 mg/day** **n = 179** **n (%)**	**Placebo (PALI)** **n = 95** **n (%)**	**Risperidone** **2-4 mg/day** **n = 113** **n (%)**	**Risperidone** **4-6 mg/day** **n = 129** **n (%)**	**Placebo** **(RIS)** **n = 122** **n (%)**	**Paliperidone ER 6-12 mg/day vs Risperidone** **2-4 mg/day**	**Paliperidone ER 6-12 mg/day vs Risperidone** **4-6 mg/day**

**Placebo-Adjusted AEs More Common With Paliperidone ER Than Risperidone**							

Insomnia	26 (14.5)	9 (9.5)	25 (22.1)	22 (17.1)	23 (18.9)	NA^†^	5.0

Sinus tachycardia	14 (7.8)	4 (4.2)	1 (0.9)	2 (1.6)	0 (0.0)	2.7	2.0

Tachycardia	12 (6.7)	3 (3.2)	1 (0.9)	3 (2.3)	0 (0.0)	2.6	NA^†^

**Placebo-Adjusted AEs More Common With Risperidone Than Paliperidone ER**							

Somnolence	7 (3.9)	5 (5.3)	10 (8.9)	9 (7.0)	2 (1.6)	7.3	5.4

Restlessness	0 (0.0)	0 (0.0)	9 (8.0)	7 (5.4)	1 (0.8)	7.2	4.6

Nausea	4 (2.2)	4 (4.2)	9 (8.0)	11 (8.5)	4 (3.3)	4.7	5.2

Anxiety	3 (1.7)	2 (2.1)	11 (9.7)	14 (10.9)	7 (5.7)	4.0	5.2

Salivary hypersecretion	3 (1.7)	0 (0.0)	6 (5.3)	5 (3.9)	0 (0.0)	3.6	2.2

Akathisia	8 (4.5)	3 (3.2)	5 (4.4)	6 (4.7)	1 (0.8)	2.3	2.6

Dizziness	8 (4.5)	3 (3.2)	5 (4.4)	8 (6.2)	3 (2.5)	NA^†^	2.4

Nasal congestion	1 (0.6)	0 (0.0)	4 (3.5)	7 (5.4)	3 (2.5)	NA^†^	2.3

*If Δ ([risperidone-placebo (RIS)] – [paliperidone-placebo (PALI)]) ≥2%. If the placebo rate for any adverse event (AE) was greater than for active treatment, then active treatment minus placebo was set equal to 0.

^†^Signifies placebo-adjusted AEs <2%.

All AEs were those reported by week 6.

AE = adverse event; NA = not applicable

Weight change was significantly greater with paliperidone ER, risperidone 2-4 mg/day and risperidone 4-6 mg/day than with placebo (all p values < 0.001), but was not significantly different among the active-treatment groups. Adjusted mean change (SE): paliperidone ER, 0.7 (0.3) kg; risperidone 2-4 mg/day, 1.0 (0.4) kg; and risperidone 4-6 mg/day, 1.3 (0.4) kg (paliperidone ER vs risperidone 2-4 mg/day, p = 0.487; vs risperidone 4-6 mg/day, p = 0.235).

## Discussion

This analysis used propensity score matching of subjects from randomized placebo-controlled schizophrenia studies to compare paliperidone ER 6-12 mg/day with risperidone 2-4 mg/day and risperidone 4-6 mg/day. Although the approved dose range of paliperidone ER is 3-12 mg/day, this analysis focused on 6-12 mg/day because comparisons between paliperidone ER 6-12 mg/day and risperidone 2-4 mg/day were expected to provide similar systemic drug exposure based on pharmacokinetic data [2]. Comparisons between paliperidone ER 6-12 mg/day and risperidone 4-6 mg/day were performed as these dose ranges were expected to yield the most favorable risk/benefit ratios based on clinical data.

The significant difference in the mean PANSS total scores suggested that paliperidone ER 6-12 mg/day may be more efficacious than risperidone 2-4 mg/day. Consistent results were observed on PANSS factor scores, CGI-S scores, response rates and placebo-corrected discontinuation rates for lack of efficacy. Data further suggested that paliperidone ER 6-12 mg/day achieved good overall tolerability compared with risperidone 2-4 mg/day, except for increased rates for tachycardia and sinus tachycardia. Discontinuation rates due to AEs were comparable, and weight gain for the 2 groups was similar.

Changes in the mean PANSS total score suggested that paliperidone ER 6-12 mg/day may be similar to risperidone 4-6 mg/day in terms of efficacy. This result is consistent with PANSS factor score and response rate data; however, overall clinical status (CGI-S) improved significantly more with paliperidone ER. Also, these data suggest that with the exception of increased rates of insomnia and tachycardia, paliperidone ER 6-12 mg/day achieved good overall tolerability compared with risperidone 4-6 mg/day.

Because there is no control over treatment assignments, covariate differences between groups may lead to biased estimates of treatment effects, as treatment groups may not be comparable. The advantage of propensity score matching is that observed covariates between groups can be balanced, thereby reducing selection bias for treatment assignment [14]. Further, this analysis used individual rather than group data, offering advantages over meta-analytic techniques such as the ability to identify the exact populations to be studied and to have access to individual data points. Notably, although studies were conducted at different times and in different countries, no significant differences were found in baseline-to-end point change on any efficacy measure between the placebo groups from the paliperidone ER and risperidone studies, suggesting that identification of analysis populations using this approach was viable for comparing paliperidone ER and risperidone.

However, propensity score analyses have several limitations. First, because this analysis relies on clinical trial databases, differences in trial design between the paliperidone ER and risperidone studies may have introduced additional bias in estimating treatment differences. The shorter duration of RIS-USA-72 (4 weeks vs 6-8 weeks for all other studies) (Table 1) could have introduced a bias favoring risperidone, particularly with regard to completion rates and AE reports. Further, entry into open-label extensions of the paliperidone ER studies was permitted at week 3; in the RIS-INT-3 risperidone study, however, subjects could enter the open-label extension only if they completed the double-blind phase. This difference could have introduced a bias for completing the risperidone study. At the time the risperidone studies were conducted, regulations limited inclusion of women in phase III clinical trials, resulting in marked differences in the percentages of women included in the risperidone and paliperidone studies. Additionally, as patient-level data are necessary to perform propensity score analyses, this method can only be used if individual patient-level data are available.

A literature search (through December 31, 2009) identified 4 additional placebo-controlled studies of paliperidone ER monotherapy and 5 additional placebo-controlled studies of risperidone monotherapy that were not included in the present analysis. Individual patient-level data were available for the 4 paliperidone ER studies and for 1 of the risperidone studies, but their designs were inappropriate for inclusion. The study by Tzimos et al examined subjects that were >65 years old [15]. The study by Luthringer et al was 2 weeks in duration and focused on the effect of paliperidone ER on sleep measurements [16]. The study by Kramer et al focused on relapse prevention; it included subjects who were initially stabilized on paliperidone ER for 8 weeks prior to the double-blind phase [17]. The paliperidone ER study by Canuso et al and the risperidone study by Potkin et al evaluated monotherapy for only 2 weeks, followed by a 4-week additive therapy phase during which additional psychotropics could be administered [18,19]. For the remaining risperidone studies [20-23], individual patient-level data for the propensity score analysis were not available.

Because the propensity score matching approach does not include all of the subjects from the original studies, differences can arise in the patient populations. The nonrandomized design of this study can limit the clinical interpretation of these results. One example is the significant difference in placebo completion rates—36.8% for the placebo (PALI) group vs 51.6% for the placebo (RIS) group—which influenced the placebo-adjusted response rate of the risperidone groups. Additionally, the risperidone 2-4 mg/day did not separate from placebo on PANSS total score change and therefore may not have been a valid active risperidone comparison for paliperidone ER 6-12 mg/day. However, the original risperidone study found that the 2 mg/day dose was superior to placebo [12]. In fact, the p value (0.052) in this analysis only narrowly missed statistical significance. As a result, the significant differences in PANSS total scores between the paliperidone ER 6-12 mg/day and risperidone 2-4 mg/day groups will need to be confirmed using randomized controlled studies to establish their clinical relevance. Additionally, the dropout rates in studies need to be considered when interpreting the efficacy findings. Although it is easy to implement the LOCF methodology, this method may not be the most robust approach in estimating the true treatment differences and controlling type I error rates. Also, in this particular analysis these trials were of a short duration, as is generally found with placebo-controlled trials in schizophrenia, and long-term effectiveness of these dosage groups could therefore not be evaluated.

Comparison of risperidone and paliperidone ER prolactin levels was limited by the availability of data and differences in the timing of specimen collection and trial duration. Prolactin specimens were collected in only 1 risperidone study (RIS-INT-3) vs all 3 paliperidone ER trials. The blood samples in the paliperidone ER studies were obtained at T_max_, whereas the timing of blood draws was not specified in the risperidone study. Further, prolactin data for risperidone were available only at baseline and at week 8 end point, prohibiting comparisons at the same time point (week 6) between risperidone and paliperidone ER. However, an analysis from a separate 6-day phase I study in stable subjects with schizophrenia found similar prolactin pharmacokinetic profiles (C_max _and AUC) when subjects received the highest recommended dose of paliperidone ER (12 mg/day) compared with an average dose of risperidone (4 mg/day) [2]. Finally, our ability to assess EPS severity was limited because the studies did not use the same movement disorder rating scales. With the exception of spontaneously reported akathisia, EPS-related AEs (parkinsonism, dystonia, tremor, hypertonia and hypokinesia) did not meet the criteria specified in the Methods section (AE differential ≥ 2%), which identified notable differences in AE rates between paliperidone ER and risperidone.

## Conclusions

In the absence of prospective, randomized, head-to-head clinical trials, a statistical comparison using propensity score matching of pooled data may be a feasible and informative technique to provide a preliminary comparison of 2 medications. This analysis suggests that paliperidone ER 6-12 mg/day may be as efficacious as risperidone 4-6 mg/day and more efficacious than risperidone 2-4 mg/day. AE-adjusted incidence rates found differences between the treatment groups that may be relevant for individual patients. As this was an indirect analysis of these 2 medications, randomized, well-controlled, head-to-head studies are required to confirm these findings.

## Competing interests

Mr. Turkoz and Dr. Canuso are full-time employees of Johnson & Johnson Pharmaceutical Research and Development, LLC, and are Johnson & Johnson stockholders. Dr. Bossie is a full-time employee of Ortho-McNeil Janssen Scientific Affairs, the company that funded the research, and is a Johnson & Johnson stockholder. Dr. Lindenmayer has received grant/research support from Janssen, Eli Lilly and Company, AstraZeneca, Johnson & Johnson, Pfizer, Bristol-Myers Squibb, Otsuka and Dainippon Sumitomo; and has served as a consultant to Janssen, Eli Lilly and Company and Schering-Plough. Dr. Schooler has received grant/research support from AstraZeneca, Bristol-Myers Squibb, Eli Lilly and Company, H. A. Lundbeck, Ortho-McNeil Janssen and Pfizer Inc; and has served as a consultant/advisory board member/speaker for Dainippon Sumitomo, Eli Lilly and Company, H. A. Lundbeck, Organon, Ortho-McNeil Janssen and Schering-Plough.

## Authors' contributions

IT, CB and CC contributed to the conception and design, acquisition of data, analysis and interpretation of data and drafting of the manuscript and its critical revision for important intellectual content. JPL and NS were involved in the interpretation of data, and critical drafting and revising of the manuscript for important intellectual content. All authors read and approved the final manuscript.

## Pre-publication history

The pre-publication history for this paper can be accessed here:

http://www.biomedcentral.com/1471-244X/11/21/prepub
